# Abnormal expression of fission and fusion genes and the morphology of mitochondria in eutopic and ectopic endometrium

**DOI:** 10.1186/s40001-023-01180-w

**Published:** 2023-07-01

**Authors:** Chaoshuang Ye, Pei Chen, Bingning Xu, Yang Jin, Yongchao Pan, Tianyu Wu, Yongjiang Du, Jingxia Mao, Ruijin Wu

**Affiliations:** Department of Gynecology, Women’s Hospital, Zhejiang University School of Medicine, Key Laboratory of Women’s Reproductive Health of Zhejiang Province, Hangzhou, 310006 China

**Keywords:** Endometriosis, Mitochondria, Fission, Fusion, Mitochondrial morphology, Cristae

## Abstract

Mitochondria play a pivotal role in physiological and metabolic function of the cell. Mitochondrial dynamics orchestrate mitochondrial function and morphology, involving fission and fusion as well as ultrastructural remodeling. Mounting evidence unravels the close link between mitochondria and endometriosis. However, how mitochondrial architecture changes through fission and fusion in eutopic and ectopic tissues of women with ovarian endometriosis remains unknown. We detected the expression of fission and fusion genes and the morphology of mitochondria in eutopic and ectopic endometrium in ovarian endometriosis. The results showed that the expression of *DRP1* and *LCLAT*1 was upregulated in eutopic endometrial stromal cells (ESCs), and the expression of *DRP1*, *OPA1*, *MFN1*, *MFN2*, and *LCLAT1* was significantly downregulated in ectopic ESCs, and reduced number of mitochondria, wider cristae width and narrower cristae junction width was observed, but there was no difference in cell survival rate. The altered mitochondrial dynamics and morphology might, respectively, provide an advantage for migration and adhesion in eutopic ESCs and be the adaptive response in ectopic endometrial cells to survive under hypoxic and oxidative stress environment.

## Introduction

Endometriosis is a chronic benign disease defined as the presence of endometrial tissue outside the uterine cavity, which causes pelvic pain and infertility, imposing both physical and psychological impacts on women of reproductive age [1, 2]. The etiopathogenesis of endometriosis has not yet been clearly elucidated. The most widely accepted concept is Sampson’s hypothesis of retrograde menstruation [3, 4], which postulates that regurgitated endometrial debris during menstruation adheres to the peritoneum and the ovary and forms lesions. Recent studies have shaped the idea that altered functionality of mitochondria might contribute to the occurrence and development of endometriosis [5–7].

Mitochondria are classically considered as ‘powerhouses of the cell’ due to their efficient capacity for ATP generation. Mitochondrial dynamics is essential for maintaining adequate energy balance through continuous fission and fusion [8]. The main dynamin-related GTPases involved in the process of mitochondrial fission and fusion in mammals are dynamic-related protein 1 (*DRP1*), mitofusin1 and 2 (*MFN1* and *MFN2*), and optic atrophy 1 (*OPA1*). *DRP1* composes the core component of the mitochondrial fission process while *MFN1/2* and *OPA1* are, respectively, responsible for outer and inner mitochondrial membrane fusion. Imbalanced mitochondrial dynamics leads to numerous diseases especially neurodegenerative diseases and cancer [9–11]. It has been shown that excessive mitochondrial fission impedes endometrial stromal cell (ESCs) migration and induced ESCs apoptosis [6]. However, little is known about how the mitochondrial fission and fusion change in endometrium of women with endometriosis. Lysocardiolipin acyltransferase 1 (*LCLAT1*) is an acyltransferase tightly linked with mitochondrial dysfunction [12, 13] by catalyzing remodeling of cardiolipin. Ablation or inhibition of *LCLAT1* mitigates mitochondrial fragmentation induced by hypoxia and oxidative stress [14]. *LCLAT1* has been studied in numbers of human diseases including obesity, cardiovascular diseases, and Parkinson’s diseases [12, 13, 15, 16], but whether *LCLAT1* plays a role in endometriosis remains unknown.

The structure of the mitochondria is in the order of the outer mitochondrial membrane (OMM), intermembrane space, inner mitochondrial membrane (IMM), and matrix from the outside to the inside. The IMM protrudes into the matrix to form cristae where respiratory complexes localize. Cristae shape affects the formation and assembly of respiratory chain super-complexes (SCs) independently of mitochondrial DNA (mtDNA) copy number and mtDNA translation [17], thereby affecting respiratory function. A series of studies have revealed abnormal cristae morphology in plenty of diseases such as pancreatic cancer [18], neurodegenerative diseases [19, 20], and ovarian cancer [21].

Here we studied the expression of genes related to mitochondrial fission and fusion, mitochondrial ultrastructure, and cell survivability in eutopic and ectopic ESCs of women with endometriosis, aiming to explore the changes of mitochondrial dynamics in endometriosis.

## Materials and methods

### Human participants and tissue acquisition

Ethical approval was obtained from the Ethics Committee of Women's Hospital, School of Medicine, Zhejiang University. One hundred and twenty-nine women who had regular menstrual cycles and undergone surgical treatment in Women's Hospital from July 2019 to February 2021 were enrolled in this study and signed written informed consent before being recruited. There were 58 cases of ectopic endometrial tissues (EC) from patients (mean age: 32.93 ± 0.86 years old) with stage III–IV endometriosis (according to revised AFS classification) and 31 cases (mean age: 33.55 ± 0.94 years old) of their homologous eutopic endometrium (EU). The controlled endometrial tissues (CE) were obtained from 71 women (mean age: 34.48 ± 1.10 years old) with other benign gynecological diseases including hydrosalpinx, mediastinal uterus, etc. All the patients were in the proliferative phase of the menstrual cycle.

Patients with hypertension, neoplastic, autoimmune diseases, malignant tumors, or other severe medical diseases were excluded. No hormone or similar drugs were used for 6 months before surgery. Differences pertaining to comparison among age and weight were not statistically significant.

### Isolation and identification of endometrial stromal cells

After rinsing with phosphate-buffered saline (PBS) and minced into 1 × 1 × 1 mm^3^ pieces, the specimens were digested with collagenase I (2 mg/ml, BioFroxx, Germany) for 60–90 min at 37 °C, 160 rpm. Then the mixture was filtered through 100 μm and 40 μm cell strainers successively, followed by resuspending the cell in DMEM/F12 medium containing 10% fetal bovine serum (BI, Israel) and incubating at 37 °C, 5% CO_2_. Antibody against vimentin (1:100, 5741, CST, USA) was used to identify the purity of primary stromal cells for its widely expressing in mesenchymally derived cells. The purity of isolated primary cells (> 95%) was identified by immunofluorescence using vimentin.

### Immunohistochemistry

Immunohistochemistry was performed according to the following procedures. Briefly, deparaffinized and dehydrated sections of endometrium were heated in a microwave oven for antigen retrieval and then rinsed in PBS. Sections were placed in 3% H_2_O_2_ for 10 min to inhibit the endogenous peroxidase activity, and then were blocked with 5% bovine serum albumin (BSA; Sigma, USA) for 30 min at 37 °C. Primary antibodies for *DRP1* (1:200, ab56788, Abcam, USA), *OPA1* (1:100, 612606, BD bioscience, USA), *MFN1* (1:500, ab57602, Abcam, USA), *MFN2* (1:200, ab56889, Abcam, USA), *LCLAT1* (1:400, ab153987, Abcam, USA) were applied at 4 °C overnight. Then each section was incubated with species-matched secondary antibodies (DAKO, Denmark) for 2 h at 37 °C. The sections were stained with diaminobenzidine (DAKO, Denmark) and counterstained with hematoxylin. Five visual fields were randomly selected in each section. The integrated optical density (IOD) and area were analyzed by Image Pro Plus 6 (https://www.mediacy.com/).

### Reverse transcription and quantitative PCR (RT-qPCR)

Total RNA was extracted with RNA extraction kit (esunbio, China) and reverse transcription was performed using reverse transcription kit (Takara, Japan). qPCR was performed using 7900HT Sequence Detection System (Applied Biosystems, CA) with PCR amplification kit (Takara, Japan). The sequence of primers used was listed as follows: 5′-TCAGTGGTGGACCTGAC-3′ (forward) and 5′-TGCTGTAGCCAAATTCGTT-3′ (reverse) for *GAPDH*, 5′-CTGCCTCAAATCGTCGTAGTG-3′ (forward)) and 5′-GAGGTCTCCGGGTGACAATTC-3′ (reverse) for *DRP1*, 5′-TGTGAGGTCTGCCAGTCTTTA-3′ (forward)) and 5′-TGTCCTTAATTGGGGTCGTTG-3′ (reverse) for *OPA1*, 5′-TGGCTAAGAAGGCGATTACTGC-3′ (forward)) and 5′-TCTCCGAGATAGCACCTCACC-3′ (reverse) for *MFN1*, 5′-CTCTCGATGCAACTCTATCGTC-3′ (forward)) and 5′-TCCTGTACGTGTCTTCAAGGAA-3′ (reverse) for *MFN2*, 5′-CACCCTACCTGTGGCATTATTG-3′ (forward)) and 5′-CCATTCTTGTCCGATGGTTCAT-3′ (reverse) for *LCLAT1*.

Experiments were carried out in triplicate wells. The relative expression levels of target genes were analyzed using the comparative 2^−ΔΔCt^ method.

### Western blot analysis

Protein was extracted using RIPA (Beyotime, China) buffer supplemented with PMSF (Beyotime, China). Protein concentration was determined by BCA assay (Beyotime, China), and added into 5 × loading buffer and followed by boiling for 10 min. Equal amounts of proteins were separated on the 12% SDS-PAGE gel, electrotransferred onto PVDF membrane (Millipore, Billerica, MA, USA), and blocked with 5% skimmed milk or 5% BSA for 1 h at room temperature. Then the membrane was incubated with the corresponding primary antibody at 4 °C overnight and incubated with the secondary antibody for 1 h at room temperature. Detection was performed with the enhanced chemiluminescence (ECL) detection reagent. Relative protein level was standardized to *GAPDH*. The antibodies included *DRP1* (1:1000, ab56788, Abcam, USA), *OPA1* (1:1000, 612606, BD bioscience, USA), *MFN1* (1:1000, ab57602, Abcam, USA), *MFN2* (1:1000, ab56889, Abcam, USA), *LCLAT1* (1:1000, ab153987, Abcam, USA), and *GAPDH* (1:5000, RK-200-301-A33, Lianke Biotech, China).

### Transmission electron microscopy (TEM)

The ultrastructure of endometrial tissues was observed with transmission electron microscopy (FEI Tecnai T10, USA, Center of Cryo-Electron Microscopy, Zhejiang University). The samples were cut into 1 × 1 × 1 mm^3^ cubes and fixed in 2.5% glutaraldehyde at 4 °C overnight. Then the samples were postfixed with 1% osmic acid at ambient temperature for 1 h and stained with uranyl acetate for 0.5 h. After dehydration with gradient ethanol and 100% acetone, samples were permeabilized for 2 h each with a 1:1 and 3:1 embedding agent/acetone at room temperature and then placed in pure embedding agent, polymerized, and sectioned with a microtome (Leica EM UC7, Vienna, Austria). Images were collected by a Tecnai 10 (FEI, 120 kV accelerating voltage) electron microscope. We observed the number and length of mitochondria, and the width of cristae junction and cristae in three groups by Image J software (https://imagej.nih.gov/ij/).

### Determination of mitochondrial mass

The mitochondrial mass was evaluated using the fluorescent dye 10-*N*-nonyl-acridine orange (NAO; A1372, Invitrogen, USA). Cells were stained with NAO (3 mM) for 30 min at 37 °C, washed with PBS, and then centrifuged at 1000 rpm for 5 min. Mitochondrial mass was further detected and analyzed using BD FACSVerse (BD Biosciences).

### Immunofluorescence

MitoTracker Green dye (Thermo Scientific, USA) was used for mitochondrial morphology analysis and further mitochondrial network analysis. Cells cultured on confocal dishes (Corning, USA) were stained with MitoTracker Green (100 nM) and Hoechst dye (Hoechst, 33342) for 30 min at 37 °C, and then observed under Fluorescence Confocal Microscopy. Mitochondrial network morphology was analyzed using MiNA plugin of Fiji software (https://imagej.net/Fiji) [22].

### Mitochondrial membrane potential (MMP)

The mitochondrial membrane potential was determined with the JC-1 mitochondrial membrane potential staining kit (Beyotime, China) using flow cytometry according to the manufacturer’s instructions. Briefly, the cells were sequentially washed with PBS, trypsinized, and incubated with 500 μL of JC-1 for 30 min at 37 °C. The precipitated cells were washed with PBS and analyzed by BD FACSVerse (BD Biosciences).

#### Statistical analysis

Experiments were performed in triplicate and at least three independent experiments. Results were calculated and expressed as mean ± SEM. One-way ANOVA was performed for statistical analysis using GraphPad Prism version 8 (GraphPad Software, CA, USA, https://www.graphpad.com). A value of *P* < 0.05 was considered statistically significant.

## Results

### Culture and identification of primary endometrial stromal cells (ESCs)

**Fig. 1:**
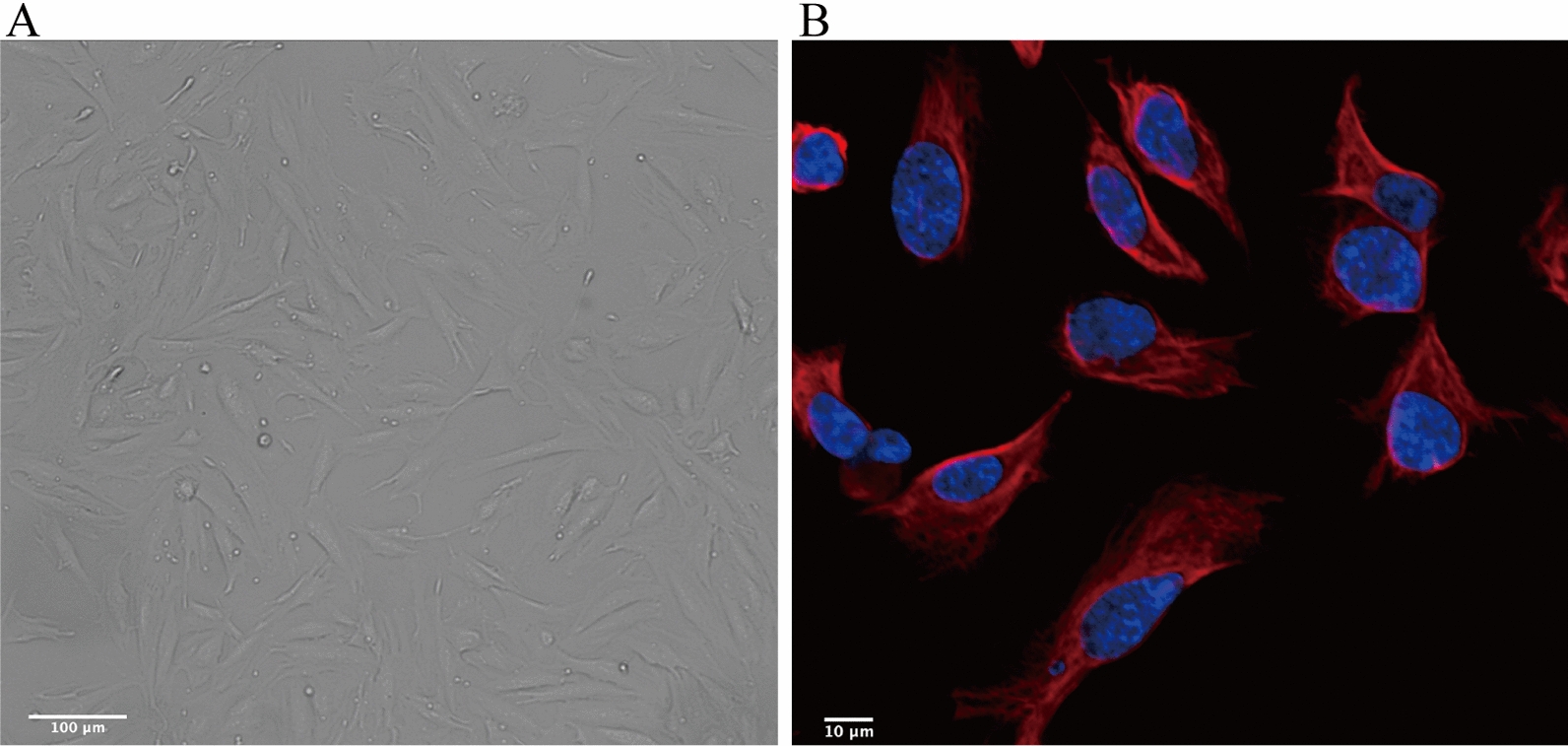
Culture and identification of ESCs in vitro. **a** ESCs were isolated from endometrial tissues and the morphology of the cultured ESCs was observed under a microscope (scale bars, 100 μm). **b** Identification of ESCs by immunofluorescent staining. ESCs were labeled with vimentin (red), and nuclear were labeled with Hoechst 33342 (blue)

The primary ESCs were isolated from endometrial tissues and ectopic endometrial cysts. The morphology of the ESCs was observed using a microscope. The cells grew adherent and exhibited a spindle or polygonal shape. Identification of the ESCs by immunofluorescence microscopy using vimentin and Hoechst 33342 showed that the purity of the stromal cells was > 95% (Fig. 1).

### Expression of molecules related to mitochondrial dynamics in ESCs

**Fig. 2:**
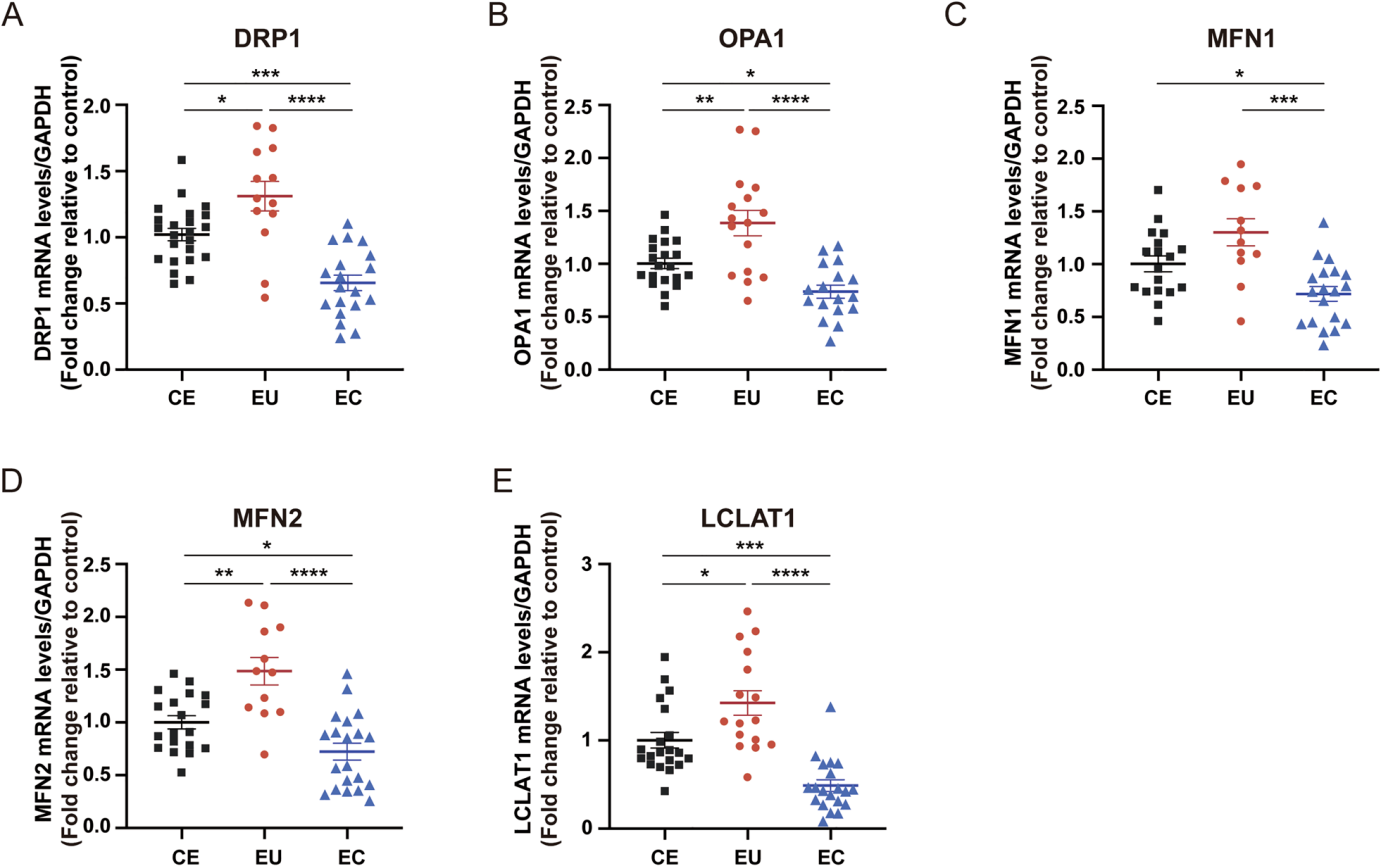
The mRNA expression of genes related to mitochondrial dynamics. Analysis of mRNA expression of *DRP1*, *OPA1*, *MFN1*, *MFN2*, and *LCLAT1* (**a**–**e**) in CE, EU, and EC by qRT-PCR (*P < 0.05; **P < 0.01; ***P < 0.001; ****P < 0.0001)

**Fig. 3:**
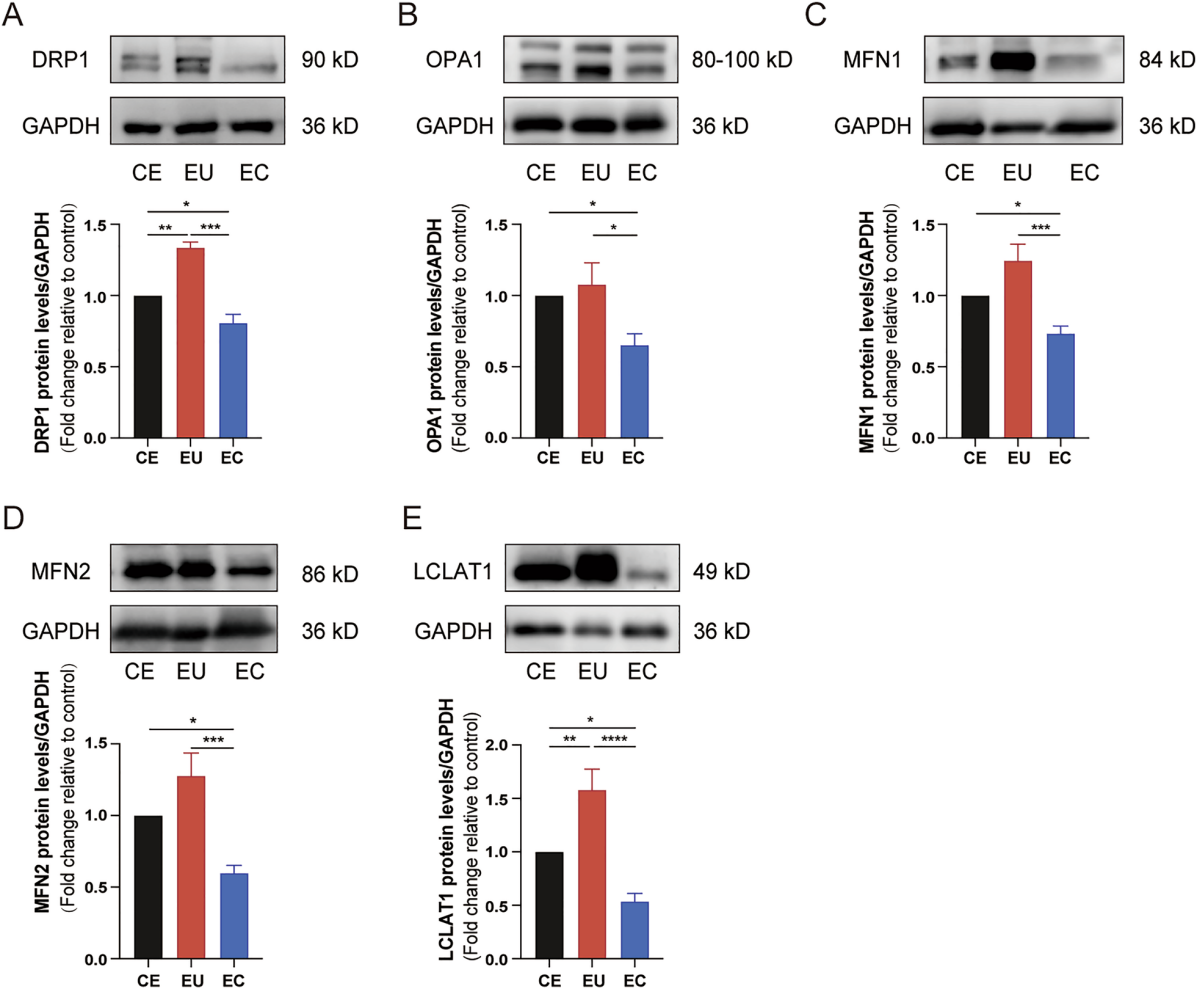
The protein expression of proteins related to mitochondrial dynamics. Analysis of protein expression of *DRP1*, *OPA1*, *MFN1*, *MFN2*, and *LCLAT1* (**A**–**E**) in CE, EU, and EC by western blotting (*P < 0.05; **P < 0.01; ***P < 0.001)

We first examined the differences in mRNA and protein expression levels of these genes in stromal cells. RT-qPCR showed that mRNA levels of *DRP1*, *OPA1*, *MFN1*, *MFN2*, and *LCLAT1* in ectopic ESCs were lower than those in controlled ESCs (*P* < 0.001, *P* < 0.05, *P* < 0.05, *P* < 0.05, *P* < 0.001, respectively) and eutopic ESCs (*P* < 0.0001, *P* < 0.0001, *P* < 0.001, *P* < 0.0001, *P* < 0.0001, respectively; Fig. 2). The mRNA expression levels of *DRP1*, *OPA1*, *MFN2*, *LCLAT1* in eutopic ESCs were increased compared with controlled ESCs (*P* < 0.05, *P* < 0.01, *P* < 0.01, *P* < 0.05, respectively; Fig. 2). Similar to mRNA levels, the protein expression levels of *DRP1*, *OPA1*, *MFN1*, *MFN2*, and *LCLAT1* in ectopic ESCs were lower than those in controlled ESCs (*P* < 0.05) and eutopic ESCs (*P* < 0.001, *P* < 0.05, *P* < 0.001, *P* < 0.001, *P* < 0.0001, respectively; Fig. 3). The protein expression levels of *DRP1* and *LCLAT1* in eutopic ESCs were increased compared with controlled ESCs (*P* < 0.01; Fig. 3). These results implicated decreased mitochondrial fission and fusion in ectopic ESCs.

### Expression of molecules related to mitochondrial dynamic in endometrial tissues

**Fig. 4:**
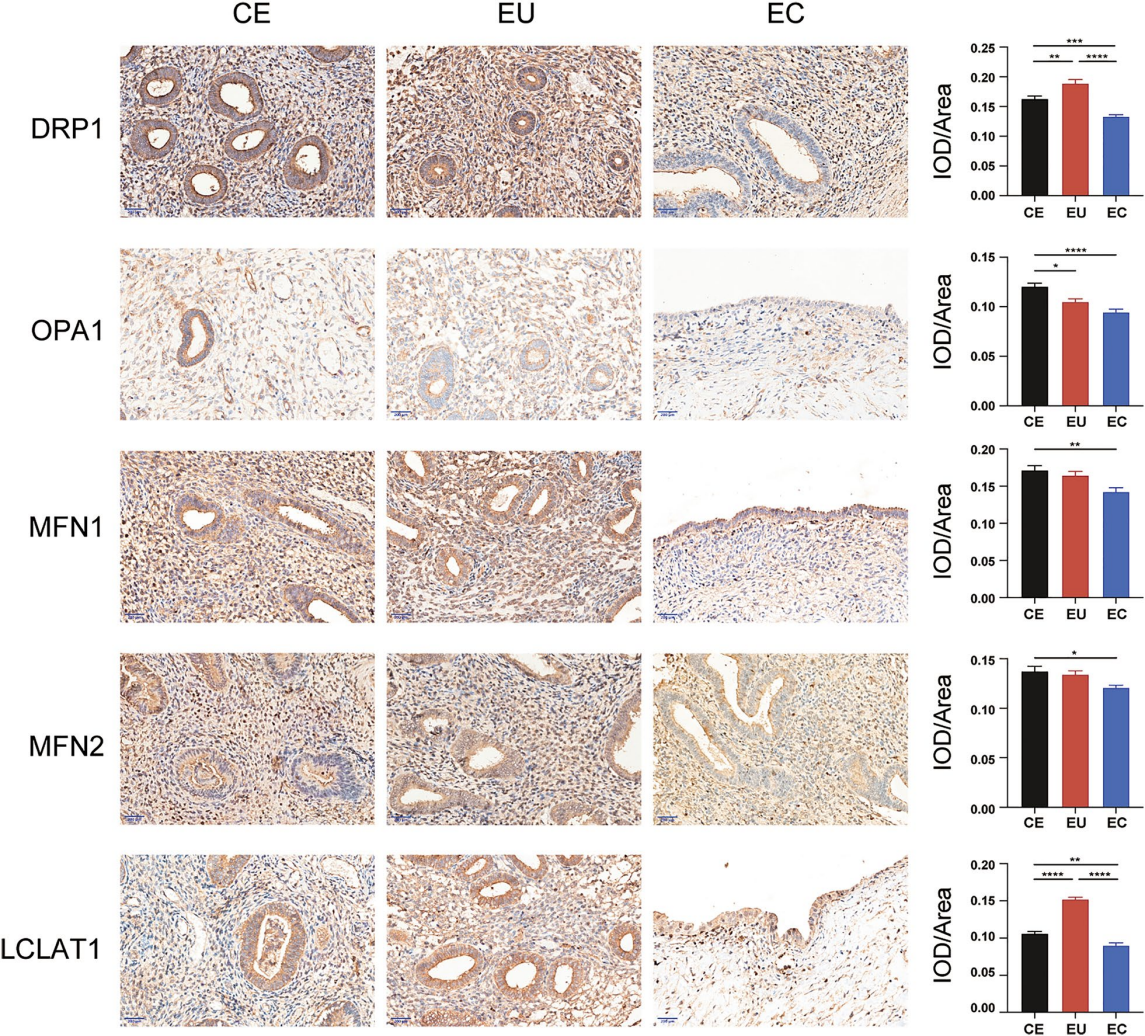
Representative images and analysis of immunohistochemical staining. Representative images and analysis of immunohistochemical staining of *DRP1*, *OPA1*, *MFN1*, *MFN2*, and *LCLAT1* in controlled, eutopic, and ectopic endometrial tissues (magnification, ×400; scale bar, 200 μm)

We further validated the expression of *DRP1*, *OPA1*, *MFN1*, *MFN2*, and *LCLAT1* in controlled, eutopic, and ectopic endometrial tissues. The proteins are distributed in the cytoplasm of the surface epithelial and stromal cells. The expression levels of *DRP1*, *OPA1*, *MFN1*, *MFN2*, and *LCLAT1* were significantly lower in ectopic endometrial tissues than in controlled endometrium (*P* < 0.001, *P* < 0.0001, *P* < 0.01, *P* < 0.05, *P* < 0.01, respectively), the expression of *DRP1* and *LCLAT1* was lower in ectopic endometrial than in eutopic endometrium (*P* < 0.0001; Fig. 4). In eutopic endometrium, the expression of *DRP1* and *LCLAT1* were higher than in controlled (*P* < 0.01, *P* < 0.0001, respectively) and *OPA1* was lower than in controlled endometrium (*P* < 0.05) (Fig. 4).

### Mitochondrial ultrastructure of ESCs under transmission electron microscopy and mitochondrial mass analysis using FACS

**Fig. 5:**
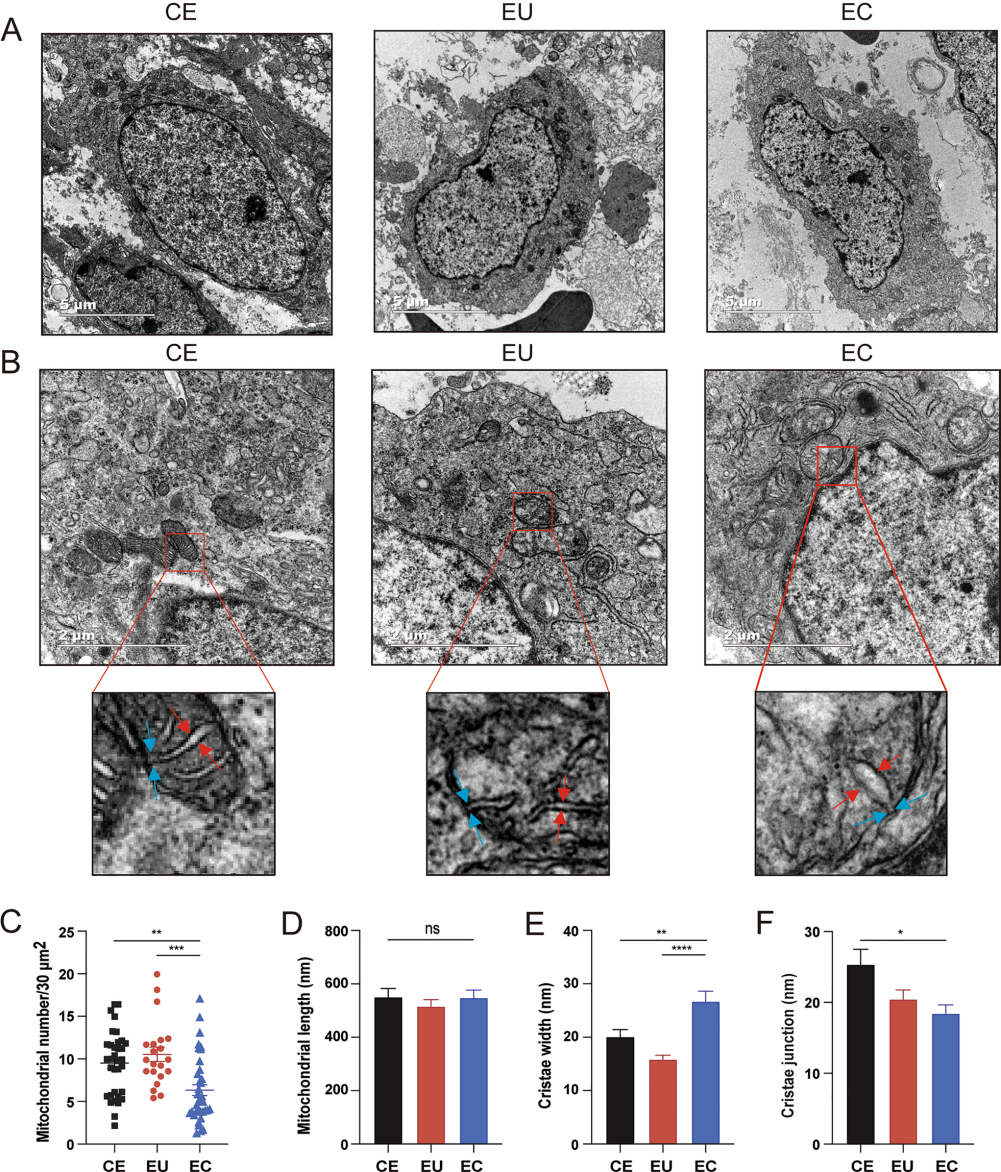
Transmission electron microscopy images of mitochondria in CE, EU, and EC. **a** Representative images of cells in CE, EU, and EC tissues (magnification, ×6800; scale bar, 5 μm). **b** Representative images of mitochondria in CE, EU, and EC tissue (23,000×; scale bar, 2 μm) and their locally magnified images. Red arrows: mitochondrial cristae width; blue arrows: width of mitochondria cristae junction. **c–f** Quantification of mitochondrial number per 30 μm^2^ (**c**), mitochondrial length (**d**), cristae width (**e**), and cristae junction width (**f**) between the three groups (*n* = 21–42 cells per group and 65–92 mitochondria per group and 45–69 cristae per group. *P < 0.05; **P < 0.01; ***P < 0.001; ****P < 0.0001)

**Fig. 6:**
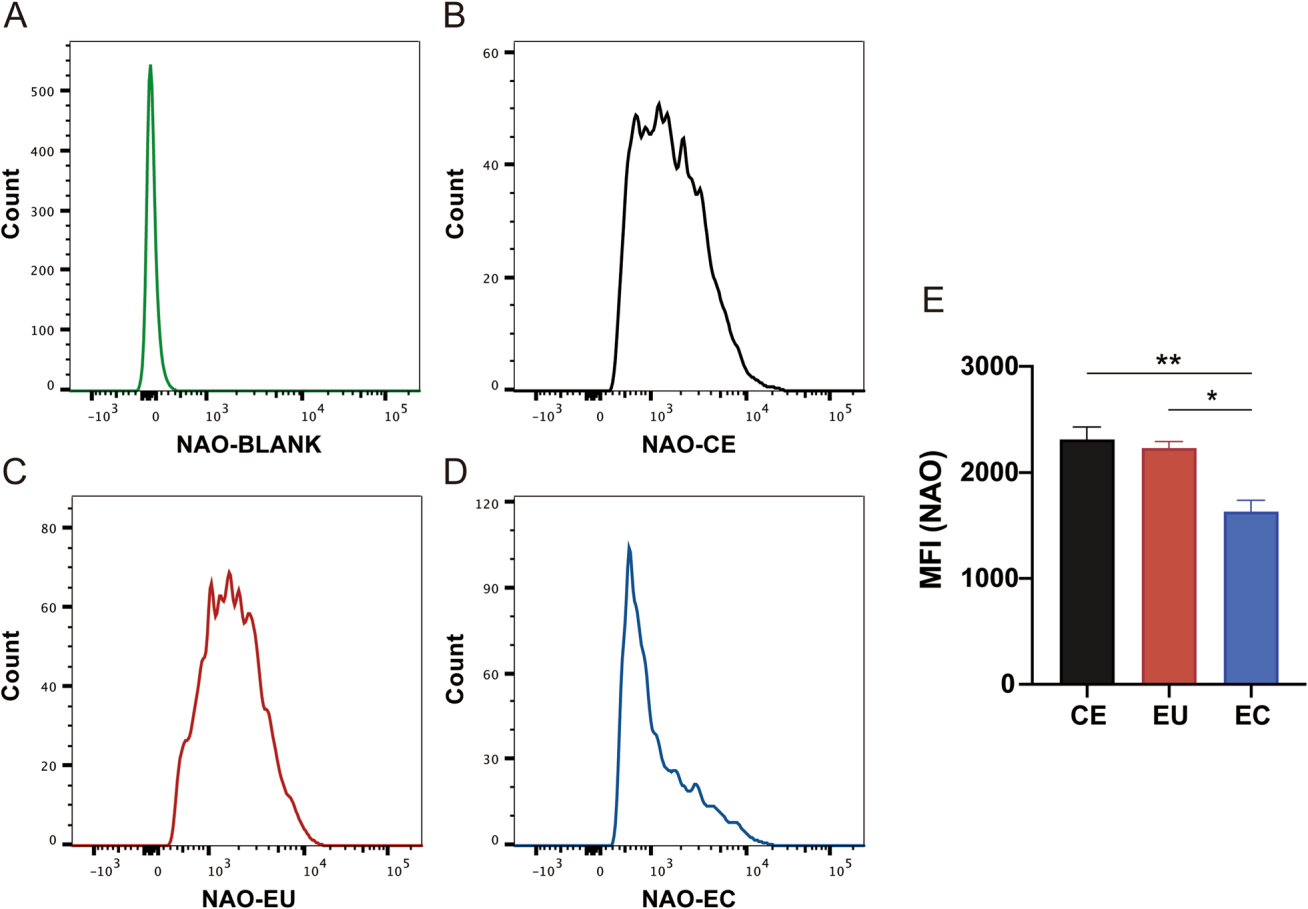
Analysis of mitochondrial mass between CE, EU, and EC using flow cytometry. The fluorescent signal was quantified by flow cytometry and is displayed as histograms (**a**–**d**) and a bar graph (**e**) (*P < 0.05; **P < 0.01)

We used transmission electron microscopy (TEM) to explore mitochondrial ultrastructure in ESCs. The results showed that the number of mitochondria in ectopic ESCs declined compared with controlled (*P* < 0.01) and eutopic ESCs (*P* < 0.001; Fig. 5C). Meanwhile, NAO staining was used to measure mitochondrial mass. The results indicated that mitochondrial mass was reduced in ectopic ESCs compared with controlled (*P* < 0.01) and eutopic ESCs (*P* < 0.05; Fig. 6). The mitochondrial cristae width in ectopic ESCs was wider than in controlled ESCs (*P* < 0.01) and eutopic ESCs (*P* < 0.0001; Fig. 5E) and the width of mitochondrial cristae junction in ectopic ESCs was narrower than in controlled ESCs (*P* < 0.05; Fig. 5F). There were no significant differences in mitochondrial length between the three groups (Fig. 5D).

### Mitochondrial network of ESCs

**Fig. 7:**
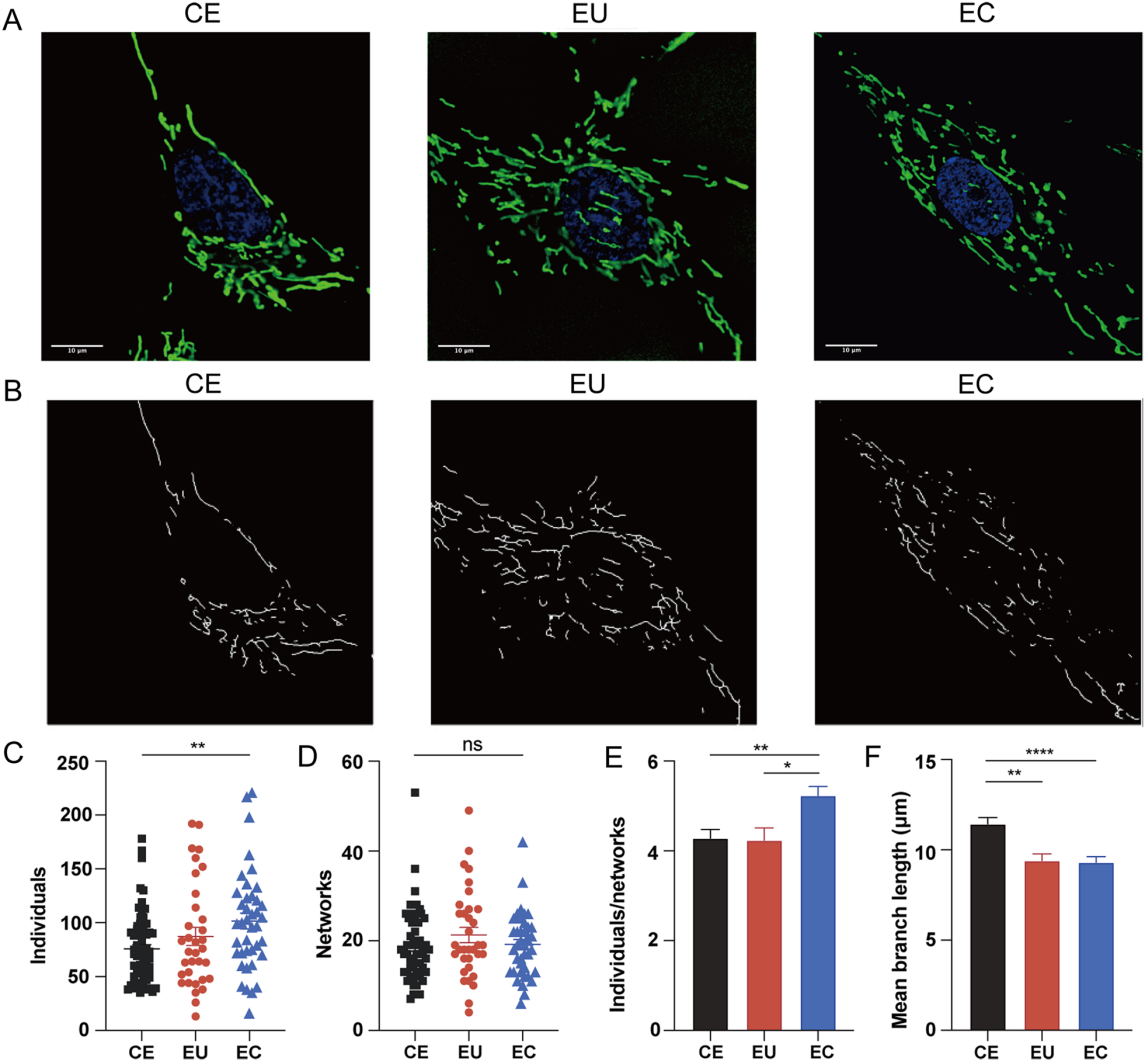
Immunofluorescence images of mitochondria in CE, EU, and EC. **a** Immunofluorescence images. **b** The processed images using MiNA plugin of Fiji software. **c**–**f** Quantitative results of individuals (**c**), networks (**d**), individuals/networks (**e**), and mean branch length (**f**) of mitochondria (*n* = 50–70 cells per group. Scale bar, 10 μm. *P < 0.05; **P < 0.01; ***P < 0.001; ****P < 0.0001)

Immunofluorescence experiments were performed in three groups. Mitochondria form a network with strong connectivity with each other, facilitating the mixing of membranes and biological materials including proteins, lipids, and other small molecules, to equilibrate the integrity of their morphology and heredity [23]. In ectopic ESCs, more individual mitochondria were observed compared with controlled ESCs (*P* < 0.01; Fig. 7C) while the number of networked mitochondria had practically no differences. The mean branch length in eutopic and ectopic ESCs was shorter than in controlled ESCs (*P* < 0.01, *P* < 0.0001, respectively; Fig. 7F). The immunofluorescence reflected an increased number of individual mitochondria with concurrent decreased mean branch length, which indicated that fragmentation of mitochondria occurred, inducing an imbalance in mitochondrial dynamics.

### Mitochondrial membrane potential (MMP)

**Fig. 8:**
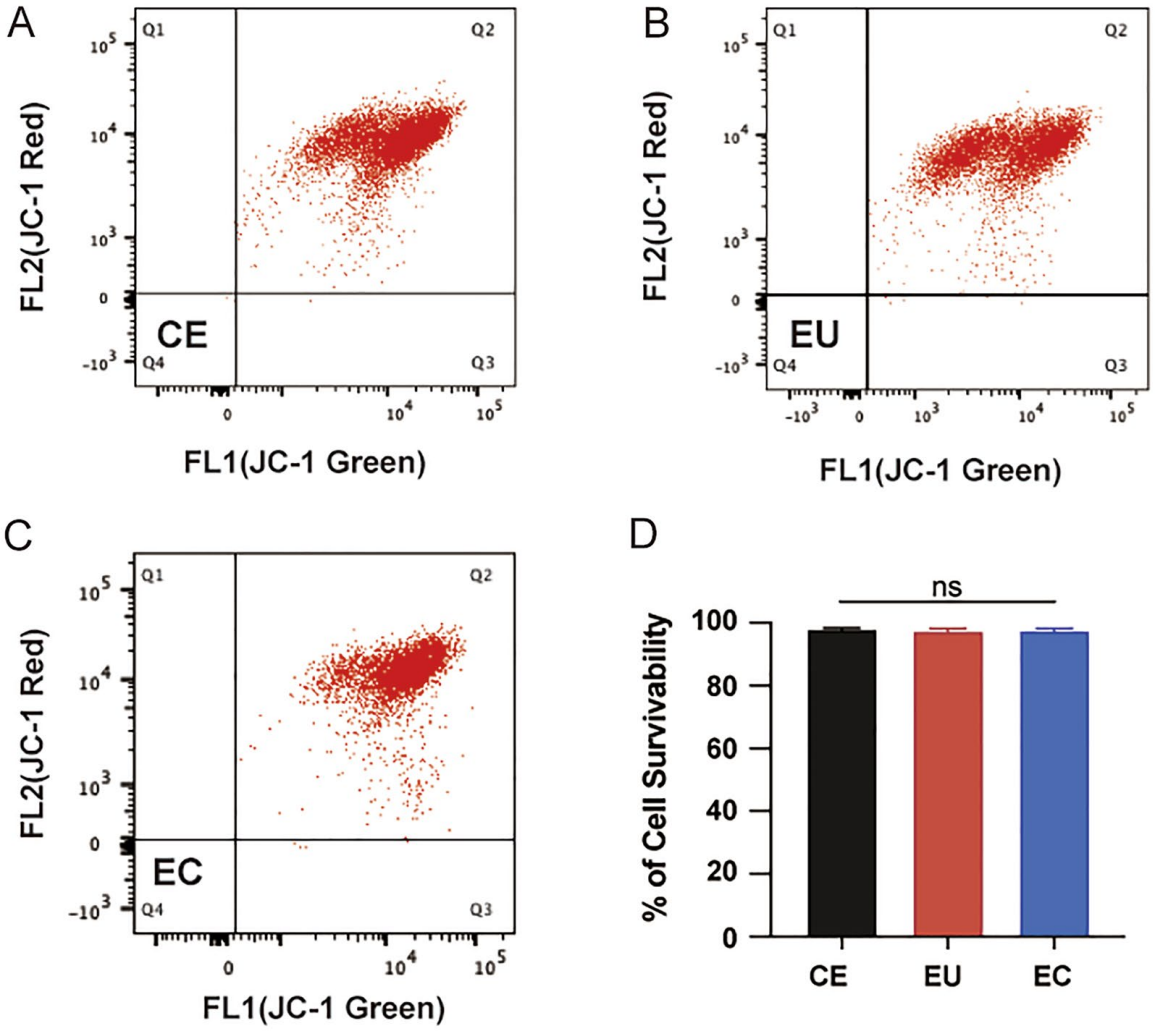
Mitochondrial membrane potential. Mitochondrial membrane potential of CE (**a**), EU (**b**), and EC (**c**) and statistic graph (**d**)

We analyzed the MMP as an effective parameter of cell survivability [24]. There were no significant differences in cell survivability among CE, EU, and EC groups (*P* > 0.05; Fig. 8).

## Discussion

Mitochondria are the primary energy hub for cellular activities, such as cell proliferation, growth, and differentiation. The morphology of mitochondria is dynamically modulated by fission and fusion process to adapt the cell to the energy metabolic status. Homeostasis of functional mitochondria requires a dynamic balance of biogenesis, fission, fusion, and degradation.

Existing studies have revealed disrupted mitochondrial morphology in obstetrical and gynecological diseases. In ovarian cancer, upregulation in mitochondrial fission, increased number and length of mitochondria were observed [21, 25]. In granulosa cells of patients with diminished ovarian reserve, oxidative stress injury caused increased mitochondrial volume and abnormal cristae [26]. Placental mitochondrial fusion was increased in gestational diabetes mellitus to reflect the increased need to compensate for mitochondrial damage due to placental cell stress [27]. Here, we demonstrated a decline in mRNA and protein expression levels of *DRP1*, *OPA1*, *MFN1*, and *MFN2* in ectopic ESCs and tissue and a decrease in mitochondrial number in endometriosis. This might alter the morphology and the rate at which the mitochondria change their shape by reducing the turnover of mitochondrial fission and fusion and thereby exerting a negative effect on the mitochondrial quality control mechanism [28].

It is easy to understand that ectopic ESCs grow in a hypoxic environment, which leads to restrained oxidative phosphorylation, enhanced glycolytic poise, and reduced mitochondrial biogenesis [29]. In the absence of oxygen as the electron acceptor, ROS accumulates due to the inefficient electron transfer. In addition, free hemoglobin and inflammatory cells in the retrograde flow of blood contribute to the rise in ROS [30, 31]. Increased oxidative stress and ROS accumulation have been found in serum, peritoneal fluid, and ectopic endometrial cysts in patients with endometriosis [5, 32], which can reduce the expression of *MFN1*, *MFN2*, and *OPA1 *[33], compatible with our result. In our study, *DRP1* also declined in ectopic ESCs, which is opposite to some other research. In hypoxic cells, upregulated expression of *DRP1* was typically observed [34], inducing fragmented mitochondria and cell apoptosis. Knockdown or inhibition of *DRP1* decreases ROS production and oxidative stress, and hinders release of cytochrome *c*, an intermediate in apoptosis, by inhibiting *DRP1*-mediated mitochondrial fragmentation [35, 36]. The different results may be related to the reduction in *LCLAT1* because its knockdown could reduce the high expression of *DRP1* triggered by the oxidative stress to mitigate the excessive fission, as well as rescue the low expression of *MFN1/2* to some extent [14, 33], protecting ESCs from oxidative stress and apoptosis. This might be an adaptive response of cells to oxidative stress and high ROS levels, and the underlying mechanism requires further study.

The expression of many genes relating to adhesion, migration, and angiogenesis was altered in eutopic endometrial tissues in patients with versus without endometriosis [37–39]. With downregulated expression of pro-apoptotic genes and upregulated expression of anti-apoptotic genes [40], eutopic ESCs might have improved survivability when under hypoxia. Besides, chronic inflammation induced by ectopic cysts contributes to epigenetic alternations in eutopic endometrium [41, 42]. We detected elevated *DRP1*, *OPA1*, *MFN2*, and *LCLAT1* in eutopic ESCs in patients with endometriosis than in controlled group. For *DRP1* and *LCLAT1*, the protein levels and immunohistochemical analysis were basically consistent with the mRNA levels. As a signature phospholipid of mitochondria, cardiolipin (CL) is sensitive to oxidative stress due to its multiple polyunsaturated fatty acids. Overexpression of *LCLAT1* leads to CL remodeling, responsible for higher susceptibility to oxidative stress [43] and therefore higher proinflammatory and adhesiveness under oxidative stress. It is confirmed that the adhesion and migration abilities of eutopic ESCs were increased when exposed to hypoxia compared to controlled ESCs [44]. *DRP1*-dependent mitochondrial fission has been widely considered as a regulator of cancer cell migration and invasion [45] and *LCLAT1* positively regulates *DRP1* expression. Therefore, when eutopic ESCs retrograde into the hypoxic peritoneal cavity, they are more prone to colonize and develop into ectopic lesion. For *OPA1* and *MFN2*, the protein levels had the same trend with the mRNA expression though they did not differ significantly, how mitochondrial fusion acts in ectopic ESCs remains to be investigated.

Cristae morphology determines the assembly and steady-state maintenance of respiratory chain super-complexes and, consequently, mitochondrial respiratory efficiency [46]. A reduction in cristae width is linked to enhanced respiratory function [47], whereas a decrease in cristae junction width reduces the release of apoptotic molecules [48]. In our study, we found wider cristae width and narrower cristae junction width, which may help alleviating oxidative injury and promoting the survival and proliferation of ectopic ESCs. Although we observed genes related to mitochondrial dynamics changed, mitochondrial appearance and structure did not seem to be affected in eutopic ESCs compared to controlled ESCs.

The mitochondrial membrane potential produced by proton pumps plays crucial roles in respiratory rate, ATP synthesis, and ROS production. The loss of MMP is regarded as an early indicator of apoptosis [49] and a hallmark of mitochondrial dysfunction [50]. We found increased granular or short rod-shaped mitochondria and decreased mean branch length of networked mitochondria in ectopic ESCs, suggesting that a small quantity of mitochondria might be in pathological conditions. But according to the MMP, there seems exhibited no significant differences in cell survivability. We speculated that though mitochondrial structure altered, it did not affect cell viability.

## Conclusion

Altered expression of genes allied to mitochondrial fission and fusion in eutopic ESCs in endometriosis may provide a significant advantage for colonization site formation. After ectopic foci formed, the decreased expression of genes related to mitochondrial dynamics and altered mitochondrial morphology might improve the adaptability of ectopic ESCs to the oxidative stress environment.

## Data Availability

Not applicable.
